# Deep learning-based classification of DSA image sequences of patients with acute ischemic stroke

**DOI:** 10.1007/s11548-022-02654-8

**Published:** 2022-05-23

**Authors:** Benjamin J. Mittmann, Michael Braun, Frank Runck, Bernd Schmitz, Thuy N. Tran, Amine Yamlahi, Lena Maier-Hein, Alfred M. Franz

**Affiliations:** 1 Medical Faculty, Heidelberg University, Im Neuenheimer Feld 672, 69120 Heidelberg, BW Germany; 2 Department of Computer Science, Ulm University of Applied Sciences, Albert-Einstein-Allee 55, 89081 Ulm, BW Germany; 3 Neuroradiology Section, District Hospital Guenzburg, Lindenallee 2, 89312 Guenzburg, BY Germany; 4 Department of Computer Assisted Medical Interventions, German Cancer Research Center (DKFZ), Im Neuenheimer Feld 223, 69120 Heidelberg, BW Germany; 5 Faculty of Mathematics and Computer Science, Heidelberg University, Im Neuenheimer Feld 205, 69120 Heidelberg, BW Germany

**Keywords:** Deep learning-based classification, DSA image sequences, Acute ischemic stroke, Overlooking thrombus

## Abstract

****Purpose**:**

Recently, a large number of patients with acute ischemic stroke benefited from the use of thrombectomy, a minimally invasive intervention technique for mechanically removing thrombi from the cerebrovasculature. During thrombectomy, 2D digital subtraction angiography (DSA) image sequences are acquired simultaneously from the posterior-anterior and the lateral view to control whether thrombus removal was successful, and to possibly detect newly occluded areas caused by thrombus fragments split from the main thrombus. However, such new occlusions, which would be treatable by thrombectomy, may be overlooked during the intervention. To prevent this, we developed a deep learning-based approach to automatic classification of DSA sequences into thrombus-free and non-thrombus-free sequences.

****Methods**:**

We performed a retrospective study based on the single-center DSA data of thrombectomy patients. For classifying the DSA sequences, we applied Long Short-Term Memory or Gated Recurrent Unit networks and combined them with different Convolutional Neural Networks used as feature extractor. These network variants were trained on the DSA data by using five-fold cross-validation. The classification performance was determined on a test data set with respect to the Matthews correlation coefficient (MCC) and the area under the curve (AUC). Finally, we evaluated our models on patient cases, in which overlooking thrombi during thrombectomy had happened.

****Results**:**

Depending on the specific model configuration used, we obtained a performance of up to 0.77$\mid$0.94 for the MCC$\mid$AUC, respectively. Additionally, overlooking thrombi could have been prevented in the reported patient cases, as our models would have classified the corresponding DSA sequences correctly.

****Conclusion**:**

Our deep learning-based approach to thrombus identification in DSA sequences yielded high accuracy on our single-center test data set. External validation is now required to investigate the generalizability of our method. As demonstrated, using this new approach may help reduce the incident risk of overlooking thrombi during thrombectomy in the future.

**Supplementary Information:**

The online version contains supplementary material available at 10.1007/s11548-022-02654-8.

## Introduction

### Medical background

The research towards enhanced treatment of patients with acute ischemic stroke has considerably improved the outcome and long-term prognosis for a majority of patients in the past few years [11]. While the drug-based thrombolytic therapy was the standard treatment for more than two decades [7], the use of thrombectomy, a minimally-invasive intervention technique for mechanically removing thrombi from the cerebrovasculature, has led to promising treatment results in several large clinical trials, recently [1, 9, 12, 30].

**Fig. 1:**
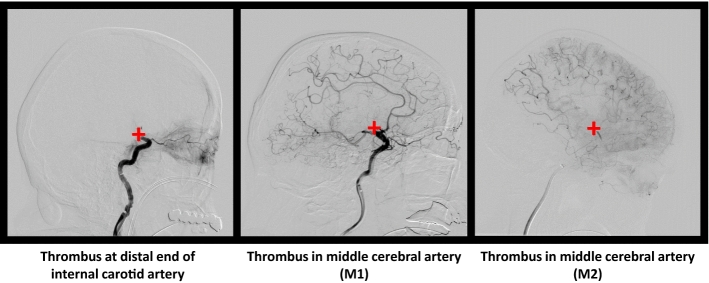
Images of DSA sequences illustrating the perfusion abnormalities caused by thrombi at different locations (proximal thrombus ending marked with a red +)

During thrombectomy, the digital subtraction angiography (DSA) is used as gold standard to visualize the cerebral blood perfusion and to control the recanalization after thrombus removal. The DSA imaging technique involves the administration of a contrast agent while simultaneously capturing a sequence of successive fluoroscopic images from the posterior-anterior (PA) and the lateral (LAT) view, forming a pair of the PA and LAT sequence, with the first shot image, serving as the mask, being digitally subtracted from all other images of the sequence. Usually, the thrombus itself is not directly visible in the images of a DSA sequence, as the contrast agent stops in front of the thrombus or flows only partially past the thrombus causing identifiable perfusion abnormalities (Fig. 1).

A serious complication during thrombectomy may be the thrombus fragmentation [17]. The main thrombus breaks into individual fragments that cause new embolisms in distal or not previously affected intracerebral-arterial areas. Based on overview DSA sequences showing the whole cerebral perfusion territory, such new occlusions can usually be directly identified and immediately be treated during the intervention. However, even though these DSA sequences are thoroughly examined by the neuroradiologists, the risk of intraprocedurally overlooking a thrombus or an embolus is increased due to several reasons: The perfusion abnormalities caused by some thrombi or emboli are difficult to detect, and the level of experience of the physicians may be relevant in this context. Furthermore, the neuroradiologists may be focused predominantly on the image area around the main thrombus. New emboli in distal intracerebral-arterial areas may therefore be missed (see *Online Resource 3* for illustrative patient cases).

How often thrombi or emboli are overlooked during thrombectomy can only be estimated, as no studies have been published on this topic. However, with rates ranging from 0% to 11% [3, 10], studies report on new intracerebral-arterial thrombi detected postinterventionally [3, 10, 22, 24]. These could be caused by distally located, intracerebral-arterial emboli that were considered as not treatable during the intervention because the risk of injury to the thin-walled distal arteries when accessing them by a catheter was too high. However, some of them could have been overlooked in the DSA sequences due to the reasons given above, as well.

One possibility to reduce the rate of such critical incidents could be provided by a software that classifies DSA sequences into thrombus-free and non-thrombus-free sequences, termed as *thrombus-yes-no-classification* in the following. Such a software could alert neuroradiologists just in time intraprocedurally if thrombi are identified. These could then possibly be treated immediately during thrombectomy in order to improve the patient’s recovery prospects.

### Related work

In contrast to a large number of publications on the automatic detection of aneurysms in DSA images [8, 16, 23] and on the thrombus detection in computed tomography images [2, 28], research results towards software-based analysis of DSA sequences of patients with acute ischemic stroke have been published only sparsely and mostly in recent years [20, 21, 25, 26].

In their feasibility study published in 2019, *Nielsen et al.* performed a *Thrombolysis In Cerebral Infarction* (TICI) [14] classification of DSA sequences by using a slightly modified ResNet18-based [13] Convolutional Neural Network (CNN), but they achieved only a poor classification performance [20]. In their follow-up publication based on a different DSA data set, they combined an EfficientNet-B0 [27] as feature extractor with a Gated Recurrent Unit (GRU) network [6] and obtained a significantly more accurate (0.95 ± 0.03) TICI classification compared to a purely EfficientNet-based approach (0.82 ± 0.02) [21]. In contrast, Schuldhaus et al. worked on automatically dividing DSA sequences into perfusion related different phases [25]. Furthermore, *Su et al.* developed an automatic, extended TICI classification scheme and reported an average area under the curve (AUC) value of 0.81 [26]. However, to the best of our knowledge, no scientific publication has reported yet on performing a thrombus-yes-no-classification.

### Contribution

We present a new deep learning-based approach to thrombus-yes-no-classification of DSA image sequences. For this purpose, we addressed the challenge of automatically identifying spatio-temporal perfusion abnormalities caused by thrombi in DSA image sequences of variable sequence length and conducted a retrospective study based on the single-center DSA data of thrombectomy patients. The study-related methods, results and discussions are described in the next sections.

## Methods

In the subsequent sections, the details regarding the medical imaging data and their annotation (“Imaging data and data annotation” section), the network architecture (“Network architecture” section), the network training routine (“Training routine” section) as well as the performed model evaluations on the test data (“Model evaluation” section) are given.

### Imaging data and data annotation

We retrospectively collected single-center DSA imaging data of 260 stroke patients ($n_{\mathrm{female}}~=~133$, $n_{\mathrm{male}}~=~127$, mean age: 74,5 years) who underwent thrombectomy in the stroke unit of the district hospital Guenzburg (BV, Germany) in the time period from January 2018 to July 2019. This resulted in a total of 1197 single DSA sequences (image resolution: $1024 \times 1024$ pixel, time resolution: 3 images/second, image sequence length: 13–61, mean: 32). The DSA sequences were acquired by the *Artis zee* cone beam CT scanner (Siemens Healthcare GmbH, Erlangen, BV, Germany).

To get our final data set, we excluded DSA sequences, when not both the PA and the LAT sequence were available ($n=8$). Furthermore, DSA sequences visualizing only the perfusion of either the vertebral arteries ($n=103$) or the external carotid artery ($n=18$) were excluded, as well. The remaining 1068 DSA sequences (534 pairs PA + LAT) were annotated by two experienced neuroradiologists. For each thrombus, they were asked to mark the proximal thrombus ending. DSA sequence pairs with no thrombus detected were classified as thrombus-free ($n=151$ pairs). Having detected a thrombus in either the PA or LAT sequence but not in the other ($n=16$), both the PA and the LAT DSA sequence were classified as non-thrombus-free. DSA sequences differing in their classification based on their annotations were re-annotated jointly by both neuroradiologists to make an ultimate decision.

Finally, we evaluated our trained models on two additional patient cases, in which overlooking thrombi during thrombectomy had happened (*Online Resource 3*).

**Fig. 2:**
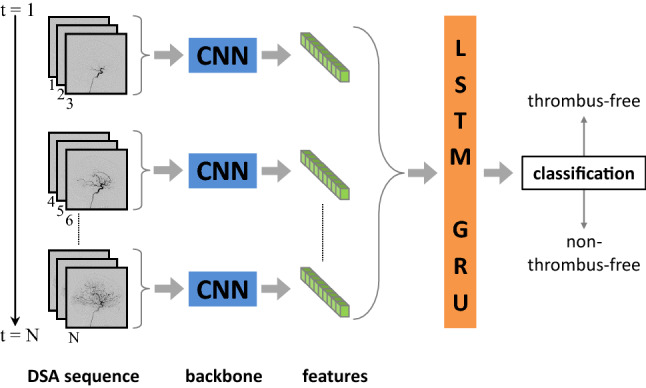
Processing a DSA sequence consisting of *N* single 2D images to be classified by the network. A CNN such as a ResNet or EfficientNet was used as feature extractor. The extracted features served as input to the LSTM or GRU network, which outputted the classification result

### Network architecture

DSA sequences are a discrete image series $D \in \{t_1 W x H, ... , t_{N}^{W x H}\}$ of temporally successive 2D images $t_{i}^{W x H}$ of varying image series length *N*, equivalent to a video of variable video length. The goal of the thrombus-yes-no-classification is to find the correct mapping between a DSA sequence *D* and a label $\hat{y} \in [0,1]$ determining whether the sequence is thrombus-free ($\hat{y} = 0$) or non-thrombus-free ($\hat{y} = 1$).

**Table 1:** CNN variants used as backbones

Network name	Source of model/weights	Pretrained on	Combined with
ResNet18	Torchvision	ImageNet	GRU
EfficientNet-B0$\mid$B1$\mid$B2$\mid$B3	Torchvision	ImageNet	GRU
EfficientNet-B0$\mid$B1	Torchvision	ImageNet	LSTM
Tf_EfficientNetV2_S$\mid$M	GitHub [29]	21k ImageNet	GRU
Rw_EfficientNetV2_S	GitHub [29]	21k ImageNet	GRU
RegNet_y_16gf	Torchvision	ImageNet	GRU

In case of the EfficientNetV2, the original tensorflow implementation (Tf_EfficientNetV2) and a slightly modified version of it (Rw_EfficientNetV2) were used, both provided by *R. Wightman* on his GitHub repository [29]

As identifying thrombi in DSA sequences is based on detecting perfusion abnormalities, the spatio-temporal contrast agent perfusion has to be analyzed to perform the classification. For this purpose, we combined a CNN as feature extractor with either a Long Short-Term Memory (LSTM) [15] network or a GRU network, that are both able to establish spatio-temporal relations between the individual images of the video sequence (Fig. 2).

To process the DSA sequence, we combined the first three single-channel grayscale images of the DSA sequence to a three-channel image, passed it through the network, prepared the following three grayscale images as next input and iterated in this way over the entire sequence. At the end of the sequence, the last image was repeatedly appended in those cases, where the sequence length was not divisible by three. Based on this approach, we were able to use pretrained weights for initializing the CNN, but the weights were not freezed during training. Before using the extracted features of the CNN as input for the LSTM/GRU network, a normalization layer and a LeakyReLu activation function were applied on the features. The bidirectional configured LSTM/GRU network consisted of 3 layers with 512 hidden units per layer and a dropout rate of 0.5 during training.

Table 1 lists the different types of CNN variants and their combination with the LSTM/GRU network used in our retrospective study. Most common, we used the GRU network, as it required less memory and trained faster compared with the LSTM network. All CNNs were modified by removing the final classification layer and adjusting the preceding average pooling layer in its pooling size such that the number of features extracted by the CNN was approximately the same for all different CNN types.

### Training routine

The first 20% of the chronologically ordered data set (“Imaging data and data annotation” section) was reserved as test data. The rest was used to train and validate the models by using five-fold cross-validation with a non-randomized train$\mid$validation split of 80%$\mid$20% for each fold. PyTorch served as deep learning library. Before feeding the DSA sequences into the network, the images were normalized and uniformly resized to $512 \times 512$ pixel to prevent out-of-memory problems on the two 24GB RAM GPUs used during training (RTX 3090 and Titan RTX, both of the NVIDIA Corporation, Santa Clara, CA, USA). Data augmentation was dynamically performed during training based on the Albumentations library [4]. The applied augmentations included vertical flipping, shift-scale-rotate transformations, changing the contrast of the images, blurring the images, adding noise and down-scaling the images to the half resolution.

**Fig. 3:**
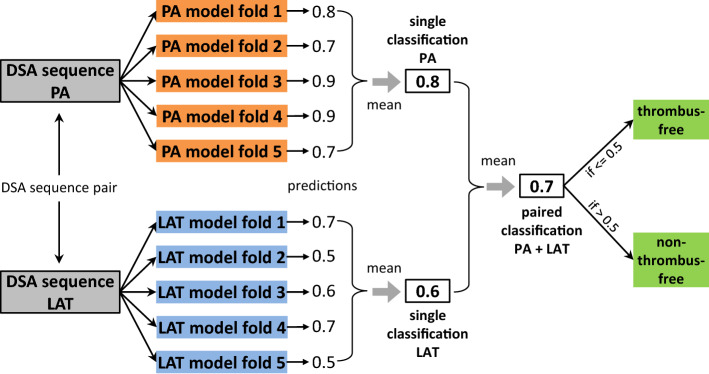
Ensembling methods used to determine the *single* and the *paired classification performance*. In both cases, the predictions were equally weighted when calculating the mean

As the PA and the LAT DSA sequence are characterized by specific perfusion structures, we trained separate models for both views, but the training routine was the same for all network variants listed in Table 1. Each model was trained end-to-end for 130 epochs by using mixed precision training [19] and binary cross entropy in its default configuration as loss function. AdamW served as optimizer [18] starting with a learning rate of $10^{-5}$, a weight decay of $10^{-2}$ and standard $\beta _1, \beta _2$. The batch size was 1, and the learning rate was reduced on plateau by a factor of $10^{-1}$. Unlike the accuracy or F1-score, the Matthews correlation coefficient (MCC) [5] does not tend to overestimate the classification performance especially for an unbalanced binary class distribution, as it was the case for our DSA data set (72% positives $\mid$ 28% negatives). Hence, we chose it as decision criterion during training and saved the model checkpoint, which achieved the highest MCC on the validation data. As we used five-fold cross-validation, we obtained five models trained on the PA sequences and five models trained on the LAT sequences (Fig. 3) for each network type listed in Table 1.

### Model evaluation

We evaluated each network variant (Table 1) regarding two aspects:

1. The *single classification performance*, i.e. classifying either the PA or the LAT DSA sequences separately, as illustrated in Fig. 3.
2. The *paired classification performance*, i.e. classifying DSA sequence pairs (PA + LAT) as a unit.

**Table 2:** Single and paired classification performance on test data set 1

Network	PA		LAT		PA + LAT	
	MCC	AUC	MCC	AUC	MCC	AUC
ResNet18 + GRU	0.58	0.90	0.54	0.88	0.58	0.92
EfficientNet-B0 + GRU	0.47	0.88	0.60	0.86	0.63	0.91
EfficientNet-B0 + LSTM	0.64	0.91	0.68	0.89	0.73	0.94
EfficientNet-B1 + GRU	0.37	0.80	0.66	0.87	0.64	0.87
EfficientNet-B1 + LSTM	0.50	0.82	0.66	0.88	0.60	0.87
EfficientNet-B2 + GRU	0.64	0.89	0.66	0.93	0.66	0.94
EfficientNet-B3 + GRU	0.38	0.86	0.61	0.90	0.64	0.91
Tf_EfficientNetV2_S + GRU	0.65	0.91	0.66	0.91	0.66	0.92
Tf_EfficientNetV2_M + GRU	0.50	0.87	0.58	0.89	0.63	0.91
Rw_EfficientNetV2_S + GRU	0.37	0.83	0.48	0.89	0.55	0.89
RegNet_y_16gf + GRU	0.59	0.89	0.44	0.84	0.49	0.90

Statistics of test data set 1: n = 102; number positives (non-thrombus-free) = 72; number negatives (thrombus-free) = 30

Furthermore, we systematically analyzed which combination of four different network variants, two for each view, resulted in the best achievable performance. Since accuracy, precision and recall used as classification metrics tend to overestimate the classification performance especially for an unbalanced binary class distribution, as it was the case for our DSA data set, we quantified the performance in terms of classification MCC (range: [-1,1]) and AUC based on the test data set. The mathematical definitions of the performance metrics are provided in *Online Resource 1*. As the test data set contained seven pairs of DSA sequences, which were subject to high annotation uncertainty due to inconspicuous, distally located, intracerebral-arterial perfusion abnormalities not treatable by thrombectomy (see example in *Online Resource 2*), we report the performance results on two variants: For test data set 1, those seven pairs were excluded, whereas they were included for test data set 2. In this way, we show how annotations that are subject to high uncertainty affect the classification performance.

## Results

The results of the single and paired classification performance of all network variants are listed in Table 2. The maximum MCC$\mid$AUC were found to be 0.65$\mid$0.91, 0.68$\mid$0.93 and 0.73$\mid$0.94 for the classification of the single PA sequences, the single LAT sequences and the pair of PA + LAT sequences, respectively.

**Fig. 4:**
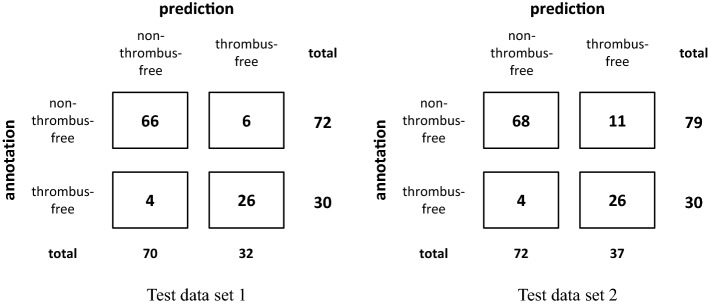
Confusion matrices corresponding to the best achievable paired classification performance on test data set 1 and 2. The corresponding MCC and AUC values are given in the text. As described above, these results were achieved by ensembling four networks, two for each view

The best classification performance with MCC$\mid$AUC values of up to 0.77$\mid$0.94 and 0.69$\mid$0.91 for test data set 1 and 2, respectively, was achieved based on a paired classification by equally ensembling four different network variants, two for each view. In this case, classifying the PA sequences was based on the combination of the ResNet18 + GRU network and the Tf_EfficientNetV2_S + GRU network, whereas classifying the LAT sequences was performed by the EfficientNet-B0 + GRU network and the EfficientNet-B0 + LSTM network. The corresponding confusion matrices of this model ensemble with respect to the performance on test data set 1 and 2 are given in Fig. 4. It should be noted that this model configuration would have correctly classified the two reported patient cases (*Online Resource 3*).

**Fig. 5:**
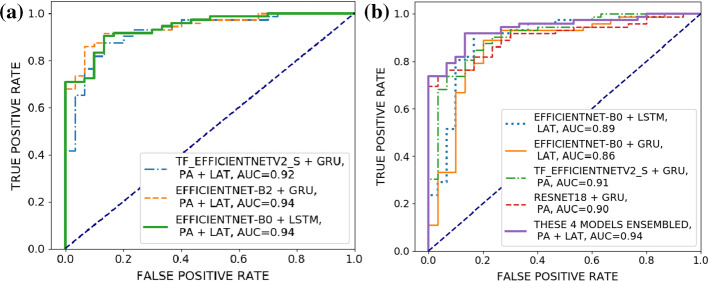
**a** ROC curves of the three network variants with the highest MCC in case of the paired classification performance. **b** ROC curve of the model achieving the best paired classification performance and consisting of four ensembled network variants. For each variant, the corresponding ROC curve is given, as well

Figure 5 shows the receiver operating characteristics (ROC) curves of selected network variants. The maximum AUC value of 0.94 was reached only when performing a paired classification. All further performance results on test data set 2 are provided in *Online Resource 2*.

## Discussion

Acute ischemic stroke highly affects the patient’s health, but the use of thrombectomy has led to promising treatment results, recently. During thrombectomy, DSA image sequences are acquired to possibly detect newly occluded areas caused by thrombus fragments split from the main thrombus. However, such new occlusions may be overlooked during the intervention. To prevent this, we developed a new deep learning-based approach to automatic thrombus-yes-no-classification of DSA sequences and found out that we could classify DSA sequences with an MCC$\mid$AUC of up to 0.77$\mid$0.94, respectively. Notably, these results were obtained on a data set with a considerably unbalanced class distribution and the ability of the networks to differentiate well between the two classes is assumed to be very good.

The observed minor drop of the classification performance in case of test data set 2 (Fig. 4) was caused by the additionally included seven pairs of DSA sequences that were subject to high annotation uncertainty. They were mostly misclassified as thrombus-free (false negative), even though they contained small, inconspicuous, distally located, intracerebral-arterial perfusion abnormalities, which, however, would not be treatable by thrombectomy or thrombolysis.

*Nielsen et al*. [20] reported an accuracy of 0.89 for classifying DSA sequences into the TICI 0 and TICI 3 class. To a limited extend, this may be regarded as equivalent to our classification performance, but we would like to point out that, contrarily to *Nielsen et al*. [21] we did not exclusively use DSA sequences with thrombi in the middle cerebral artery. Instead, our DSA sequences included thrombi at various locations inside the whole perfusion territory of the internal carotid artery as well as sequences with imaging artifacts. This diversity of affected brain regions reflects the commonly observed variety of ischemic strokes treated in stroke units, even though classifying these correctly should be regarded a challenging task.

As our retrospective study was based on single-center DSA imaging data, the trained neural networks could be less generalizable with respect to DSA data of other stroke units. Nevertheless, it is worth to note that the DSA data of the case reports (*Online Resource 3*) had a different image and time resolution compared with the training DSA data set, but these case report DSA sequences were classified correctly by the neural networks, too.

To further help the neuroradiologists in localizing thrombi, that are hard to detect, a next step to be investigated could be the thrombus detection. However, even though the annotations of our DSA data set already contained the thrombus positions, the task of automatically detecting thrombi in the DSA images should not be underestimated. In particular, this is related to the high interobserver variability of the thrombus position annotation, which was 23 ± 28 image pixels (i.e., 2% ± 3% of image width) for our DSA data set. Due to this annotation variability, it could be difficult to objectively assess the ability of a neural network to detect thrombi by using commonly evaluation metrics such as the *mean average precision*, as for this, the network predictions must be clearly categorized into right and false. Prospective studies should therefore determine how to best deal with high annotation variability and which evaluation metrics are most suitable for the thrombus detection problem.

From a medical perspective, the reached classification performance may seem acceptable, but only a prospective study can analyze how the trained neural networks will perform in clinical practice. To improve the classification performance and the generalizability, a substantially larger amount of multi-center training data would be required. Additionally, using transformer-based neural networks could be an option for improving classification performance, as well. As demonstrated by our final evaluation test on two patient cases, our deep learning-based approach could have prevented overlooking thrombi during thrombectomy. Thus, the goal of minimizing the risk of this incident may be fulfilled.

## Conclusion

Our deep learning-based approach to thrombus identification in DSA sequences yielded high accuracy on our single-center test data set. External validation is now required to investigate the generalizability of our method. As demonstrated, using this new approach may help reduce the incident risk of overlooking thrombi during thrombectomy in the future.

## Supplementary Information

Below is the link to the electronic supplementary material.

Supplementary file 1 (pdf 128 KB)

Supplementary file 2 (pdf 504 KB)

Supplementary file 3 (pdf 704 KB)

## Data Availability

Due to data protection reasons, the DSA data set cannot be published.

## Code availability

The training scripts are available on GitHub (https://github.com/BMittmannTest/DeepLearningBasedDsaClassification). The corresponding trained network models are published on the open science framework (https://osf.io/n8k4r/).
