# Supplementary material for: Deep learning-based classification of DSA image sequences of patients with acute ischemic stroke
Source: Int J Comput Assist Radiol Surg. 2022 May 23;17(9):1633–41. doi: 10.1007/s11548-022-02654-8 (PMC9463240; doi:10.1007/s11548-022-02654-8)
Supplement: Supplementary file 1 — (pdf 128 KB) [file 11548_2022_2654_MOESM1_ESM.pdf]

Definitions of classification performance metrics

The used abbreviations in this document mean the following:

- **TP:** True positive – A DSA sequence was annotated as non-thrombus-free and classified as non-thrombus-free.
- **FP:** False positive – A DSA sequence was annotated as thrombus-free but classified as non-thrombus-free.
- **TN:** True negative – A DSA sequence was annotated as thrombus-free and classified as thrombus-free.
- **FN:** False negative – A DSA sequence was annotated as non-thrombus-free but classified as thrombus-free.

The **Matthews correlation coefficient (MCC)** was defined as:

$$MCC = \frac{TP \cdot TN - FP \cdot FN}{\sqrt{(TP + FP) \cdot (TP + FN) \cdot (TN + FP) \cdot (TN + FN)}} \tag{1}$$

The **area under the curve (AUC)** was calculated based on the Python *scikit* library.

The given formula of the MCC was taken from:

Chicco D, Jurman G (2020): The advantages of the Matthews correlation coefficient (MCC) over F1 score and accuracy in binary classification evaluation. BMC Genomics 21:6. https://doi.org/10.1186/s12864-019-6413-7

Regarding the Python *scikit* library, reference is made to:

Pedregosa F, Varoquaux G, Gramfort A, Michel V, Thirion B, Grisel O, Blondel M, Prettenhofer P, Weiss R, Dubourg V, Vanderplas J, Passos A, Cournapeau D, Brucher M, Perrot M, Duchesnay E (2011): Scikit-learn: Machine Learning in Python. J Mach Learn Res, 12, 2825–2830.

Mittmann, B. J., Braun, M., Runck, F., Schmitz, B., Tran, T. N., Yamlahi, A., Maier-Hein, L., Franz, A. M.: **Deep learning-based classification of DSA image sequences of patients with acute ischemic stroke**, *Int J Comput Assist Radiol Surg*, Corresponding author: A. M. Franz (alfred.franz@thu.de), Department of Computer Science, Ulm University of Applied Sciences, Albert-Einstein-Allee 55, Ulm, 89081, BW, Germany.
