# Supplementary material for: Deep learning-based classification of DSA image sequences of patients with acute ischemic stroke
Source: Int J Comput Assist Radiol Surg. 2022 May 23;17(9):1633–41. doi: 10.1007/s11548-022-02654-8 (PMC9463240; doi:10.1007/s11548-022-02654-8)
Supplement: Supplementary file 2 — (pdf 504 KB) [file 11548_2022_2654_MOESM2_ESM.pdf]

Classification performance on test data set 2

The abbreviations used in this document are: AUC = area under the curve; LAT = lateral; MCC = Matthews correlation coefficient; PA = posterior-anterior;

Test data set 2 included a total of n = 109 DSA sequence pairs (PA + LAT), of which 79 pairs were annotated as non-thrombus-free and 30 as thrombus-free. In contrast to test data set 1, data set 2 included seven pairs of DSA sequences, which were subject to high annotation uncertainty, as they contained small, inconspicuous, distally located, intracerebral-arterial perfusion abnormalities, which, however, would not be treated by thrombectomy or by thrombolysis. An illustrative example of such a perfusion abnormality is given in Figure 1 and the corresponding case description is given below. The results of the single and paired classification performance on test data set 2 of all network variants are then listed below in Table 1.

Case description to Figure 1: In the arterial phase of the DSA sequence, no perfusion abnormality was detectable, neither in the PA nor in the LAT sequence. However, at the end of the parenchymal phase, a small minderperfused, distally located, intracerebral-arterial area (marked with a red +) was visible in the LAT sequence but not in the PA sequence. This might be caused by a very small, distally located thrombus or embolus. Hence, this DSA sequence pair was annotated as probably non-thrombus-free, even though nearly the whole perfusion territory of the internal carotid artery was perfused normally. This could be a reason for this DSA sequence pair to be misclassified by the networks as thrombus-free.

Mittmann, B. J., Braun, M., Runck, F., Schmitz, B., Tran, T. N., Yamlahi, A., Maier-Hein, L., Franz, A. M.: **Deep learning-based classification of DSA image sequences of patients with acute ischemic stroke**, *Int J Comput Assist Radiol Surg*, Corresponding author: A. M. Franz (alfred.franz@thu.de), Department of Computer Science, Ulm University of Applied Sciences, Albert-Einstein-Allee 55, Ulm, 89081, BW, Germany.

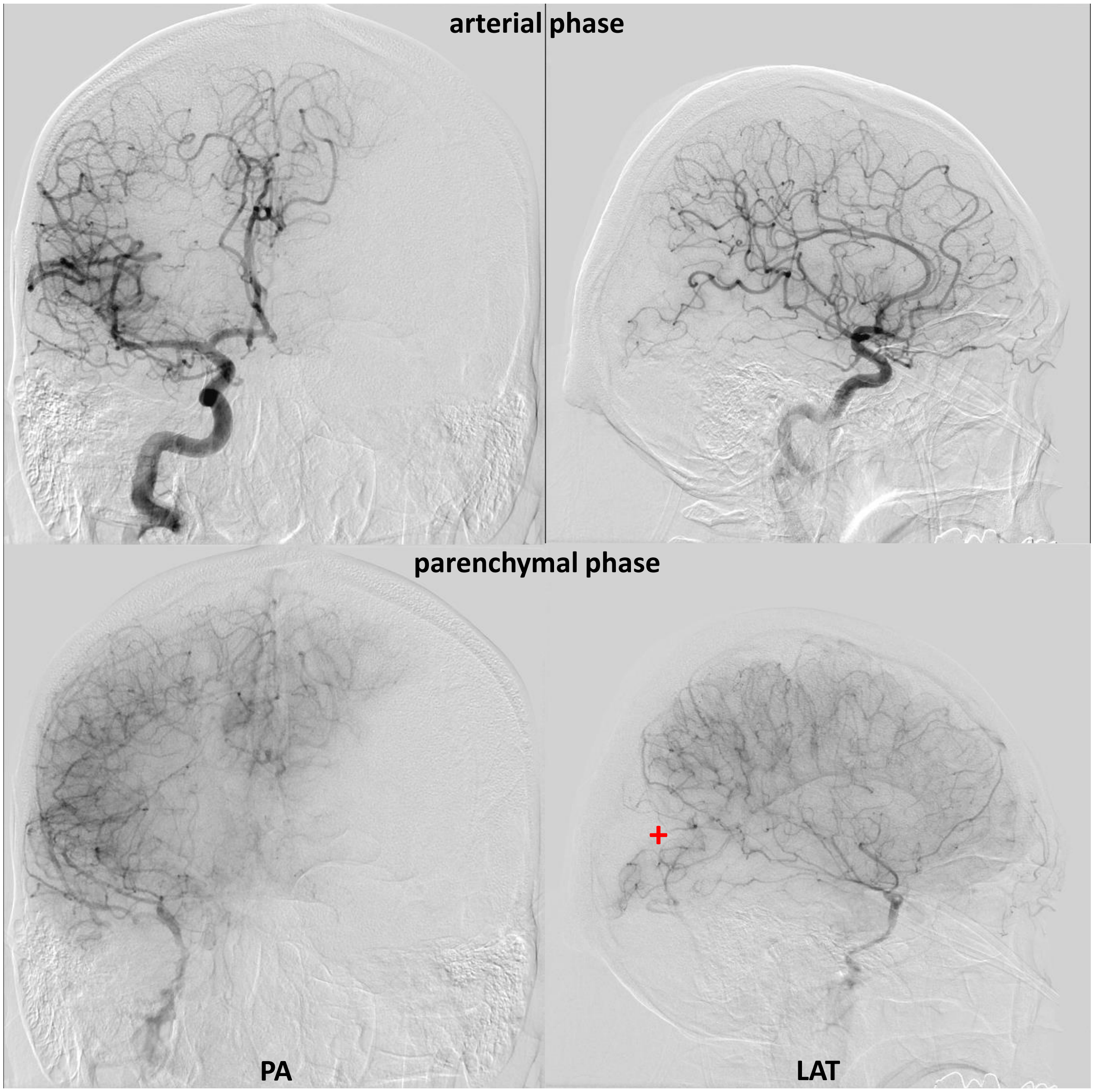

Figure 1: Illustrative example of one of the inconspicuous perfusion abnormalities in the seven pairs of DSA sequences, which were included in test data set 2 and which were subject to high annotation uncertainty. The case description is given in the text above.

Mittmann, B. J., Braun, M., Runck, F., Schmitz, B., Tran, T. N., Yamlahi, A., Maier-Hein, L., Franz, A. M.: **Deep learning-based classification of DSA image sequences of patients with acute ischemic stroke**, *Int J Comput Assist Radiol Surg*, Corresponding author: A. M. Franz (alfred.franz@thu.de), Department of Computer Science, Ulm University of Applied Sciences, Albert-Einstein-Allee 55, Ulm, 89081, BW, Germany.

Table 1: Single and paired classification performance on test data set 2.

	PA		LAT		PA + LAT	
Network	MCC	AUC	MCC	AUC	MCC	AUC
ResNet18 + GRU	0.53	**0.88**	0.49	0.85	0.52	0.89
EfficientNet-B0 + GRU	0.41	0.85	0.53	0.83	0.55	0.88
**EfficientNet-B0 + LSTM**	**0.58**	**0.88**	**0.61**	**0.86**	**0.65**	**0.91**
EfficientNet-B1 + GRU	0.33	0.78	0.60	0.85	0.56	0.85
EfficientNet-B1 + LSTM	0.46	0.79	0.60	0.85	0.56	0.85
**EfficientNet-B2 + GRU**	0.57	0.86	0.56	**0.90**	**0.59**	**0.91**
EfficientNet-B3 + GRU	0.34	0.83	0.53	**0.87**	0.57	0.88
**Tf_EfficientNetV2_S + GRU**	**0.57**	**0.88**	**0.56**	**0.87**	0.57	0.89
Tf_EfficientNetV2_M + GRU	0.46	0.85	0.47	0.85	0.55	0.88
Rw_EfficientNetV2_S + GRU	0.33	0.80	0.41	0.85	0.46	0.85
RegNet_y_16gf + GRU	0.53	0.87	0.39	0.80	0.44	0.87
