# Supplementary material for: Deep learning-based classification of DSA image sequences of patients with acute ischemic stroke
Source: Int J Comput Assist Radiol Surg. 2022 May 23;17(9):1633–41. doi: 10.1007/s11548-022-02654-8 (PMC9463240; doi:10.1007/s11548-022-02654-8)
Supplement: Supplementary file 3 — (pdf 704 KB) [file 11548_2022_2654_MOESM3_ESM.pdf]

Case reports overlooked thrombi during thrombectomy

We report two patient cases in which overlooking thrombi during thrombectomy had happened. In the first case, a thrombus in the M1 segment of the middle cerebral artery fragmented and caused a new embolism of the anterior cerebral artery. In the second case, the main thrombus was retrieved, but two distally located thrombi remained in the subsequent perfusion territory of the middle cerebral artery.

It should be noted that the DSA sequences related to the reported case reports have another image and time resolution compared with the image sequences of the DSA data set used for training, validating and testing the deep learning models. Instead of 1024 x 1024 pixel, these had an image resolution of either 1440 x 1440 or 1856 x 1856 pixel. Furthermore, instead of 3 images/second for the DSA data set, the time resolution was 2.5 images/second for these DSA sequences.

Case report 1 – thrombus fragmentation causing new embolism:
Before thrombectomy, a thrombus was located in the M1 segment of the middle cerebral artery (Figure 1). During thrombectomy, the main thrombus was retrieved, but a split thrombus fragment caused a new embolism in the anterior cerebral artery, resulting in a totally reperfused middle cerebral artery, but an occluded anterior cerebral artery (ACA). The embolus in the ACA, however, was overlooked during thrombectomy, even though it could have been treated immediately during the intervention. Postinterventionally, this led to a cerebral infarct region in the perfusion territory of the ACA.

Overlooking the ACA embolus could have been prevented with our deep learning-based approach, as our trained models would have correctly classified the corresponding DSA sequences, the pre-interventional as well as the post-interventional DSA sequences showing the embolism in the ACA.

Case report 2 – remaining distally located, intracerebral-arterial thrombi:
During thrombectomy, the main thrombus located in the M1 segment of the middle cerebral artery was retrieved, but two thrombi remained in the subsequent perfusion territory of the middle cerebral artery (Figure 2). They were overlooked during the intervention. Probably, these would not have been treated interventionally because the risk of injury to the thin-walled vessels when accessing them by a catheter would be too high. Nevertheless, knowing the presence of these thrombi is relevant regarding the post-interventional recovery process of the patient and overlooking these thrombi could have been prevented with our deep learning-based approach, as our trained models would have correctly classified the corresponding DSA sequence pair.

Mittmann, B. J., Braun, M., Runck, F., Schmitz, B., Tran, T. N., Yamlahi, A., Maier-Hein, L., Franz, A. M.: **Deep learning-based classification of DSA image sequences of patients with acute ischemic stroke**, *Int J Comput Assist Radiol Surg*, Corresponding author: A. M. Franz (alfred.franz@thu.de), Department of Computer Science, Ulm University of Applied Sciences, Albert-Einstein-Allee 55, Ulm, 89081, BW, Germany.

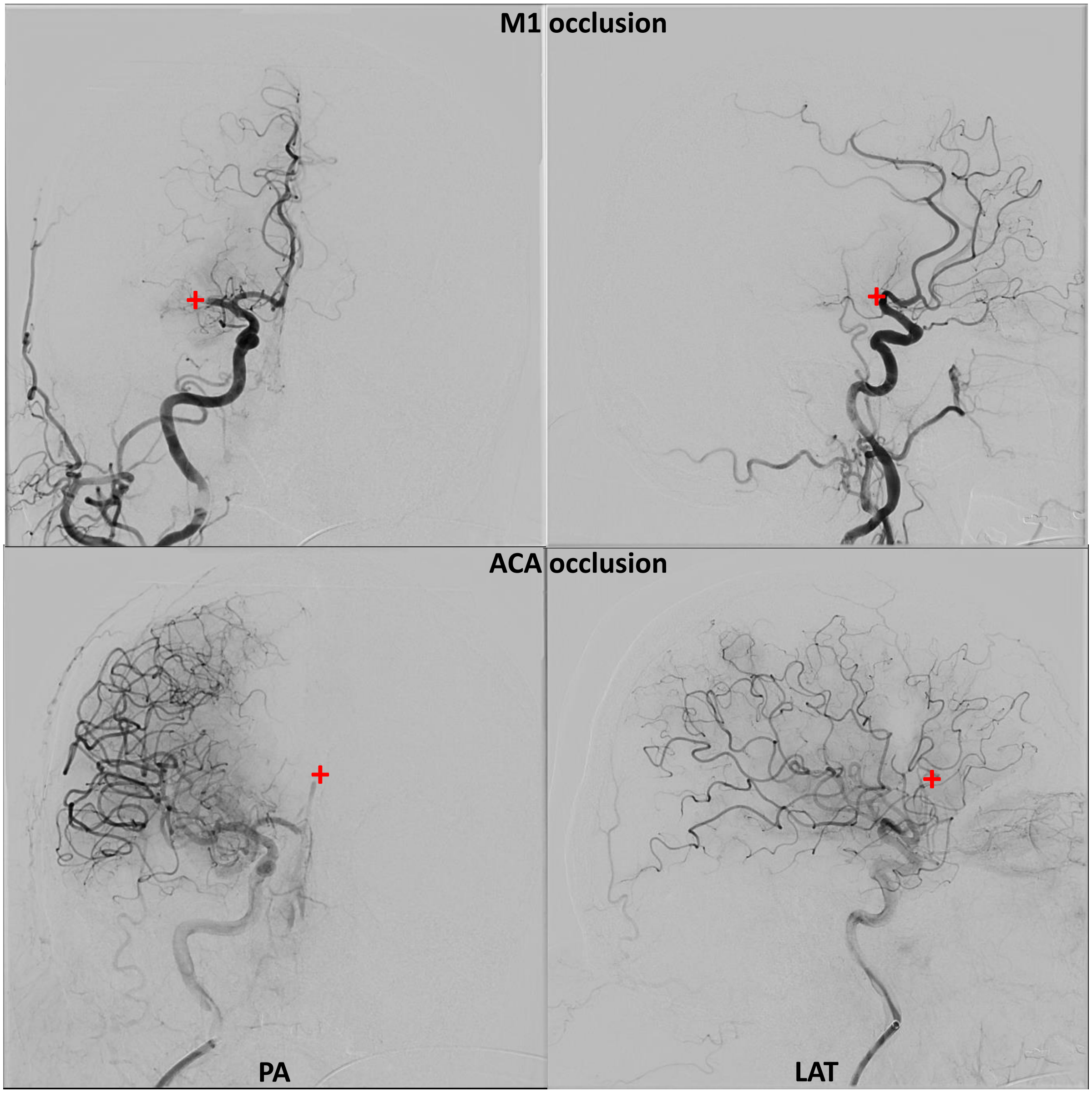

Figure 1: Case report 1 – thrombus fragmentation causing new embolism: The initial thrombus in the M1 segment (marked with red +) was retrieved, but a split thrombus fragment caused a new embolism in the anterior cerebral artery (marked with red +). The embolus in the ACA was overlooked during thrombectomy.

Mittmann, B. J., Braun, M., Runck, F., Schmitz, B., Tran, T. N., Yamlahi, A., Maier-Hein, L., Franz, A. M.: **Deep learning-based classification of DSA image sequences of patients with acute ischemic stroke**, *Int J Comput Assist Radiol Surg*, Corresponding author: A. M. Franz (alfred.franz@thu.de), Department of Computer Science, Ulm University of Applied Sciences, Albert-Einstein-Allee 55, Ulm, 89081, BW, Germany.

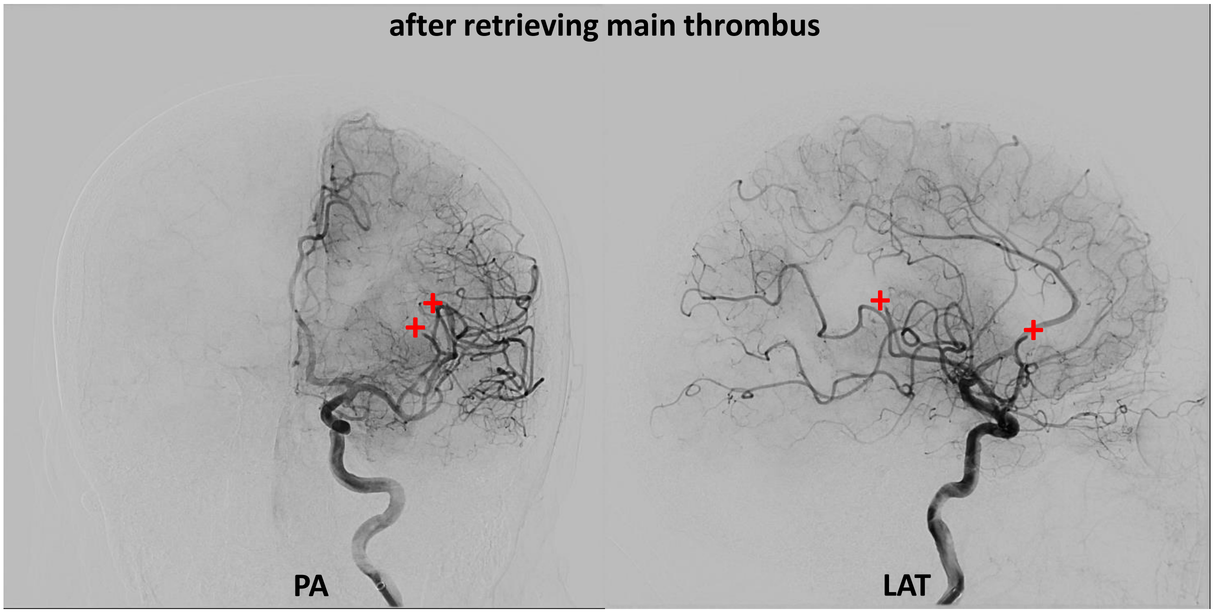

Figure 2: Case report 2 – remaining distally located, intracerebral-arterial thrombi: After retrieving the main thrombus in the middle cerebral artery, two thrombi remained in the subsequent perfusion territory of the middle cerebral artery. Both thrombi are detectable in the PA and the LAT DSA sequence (thrombus positions marked with a red +), but they were overlooked during thrombectomy.
